# Association of *BCR/ABL* transcript variants with different blood parameters and demographic features in Iraqi chronic myeloid leukemia patients

**DOI:** 10.1002/mgg3.809

**Published:** 2019-06-17

**Authors:** Mahmood S. Khazaal, Farqad B. Hamdan, Qasim S. Al‐Mayah

**Affiliations:** ^1^ Department of Physiology, College of Medicine Al‐Nahrain University Baghdad Iraq; ^2^ Medical Research Unit, College of Medicine Al‐Nahrain University Baghdad Iraq

**Keywords:** *BCR‐ABL* fusion gene variants, CML, Iraqis

## Abstract

**Background:**

Chronic myeloid leukemia (CML) is a myeloproliferative neoplasm characterized by the presence of *BCR‐ABL* fusion gene (GenBank accession NC_000022.11). In the vast majority of CML patients, the typical subtype of *BCR‐ABL* transcript are b3a2, b2a2 or both. The aim of this study was to determine the different subtypes of *BCR‐ABL* transcript and their impact on the demographic and hematological parameters in Iraqi patients with CML.

**Methods:**

One hundred patients with chronic phase CML (11 newly diagnosed and 89 imatinib‐resistant) were enrolled in this study. Ribonucleic acid (RNA) was extracted from leukocytes, and complementary DNA was created using reverse transcriptase polymerase chain reaction technique. A multiplex polymerase chain reaction with four specific primers was used to determine the *BCR‐ABL* fusion subtypes in each patient.

**Results:**

Male to female ratio was 1.38:1. Fifty‐nine patients expressed b3a2 transcript, whereas 39 of the remaining cases were positive for b2a2 variant. One case expressed b2a3 transcript, while the last case coexpressed the two subtypes of mRNA b3a2/b2a2. Male and female were significantly associated with b3a2 and b2a2 subtypes, respectively. The b3a2 subtype showed higher total leukocyte count than b2a2 subgroup, while b2a2 variant demonstrated significantly elevated platelet counts compared to those with b3a2 transcript. A significantly higher plateletcrit percentage (PCT%) was found in patients with b2a2 transcript whereas.

**Conclusions:**

The testified Iraqi group expressed M‐*BCR‐ABL* type with preponderance of b3a2 over b2a2 subtype. There was a gender‐skewed distribution in *BCR‐ABL* transcript types with b3a2 transcript more prevalent in males. The type of *BCR‐ABL* transcript is reflected by different leukocyte and platelet counts at diagnosis, which might represent a distinct phenotype and disease biology.

## 1 INTRODUCTION

Chronic myeloid leukemia (CML) is a clonal myeloproliferative neoplasm of a pluripotent stem cell characterized by the presence of Philadelphia chromosome (Ph) (Kagita, Mamidi, Digumarti, Gundeti, & Digumarti, 2018). This chromosome is a result of a reciprocal translocation between the long arms of chromosomes 9 and 22 t(9;22)(q34;q11), leading to *BCR‑ABL1* fusion gene with constitutive tyrosine kinase activity (Nowell & Hungerford, 1960). The breakpoint within the *ABL1* gene (GenBank accession NC_000009.12) is almost always at the second exon (a2), while the breakpoint in the *BCR* gene varies and can be localized to one of the three regions: major breakpoint cluster region (M‐*BCR*), minor *BCR* (m‐*BCR*), and micro‐*BCR* (Burmeister & Reinhardt, 2008; Rana et al., 2013).

Not only does the frequency of different subtypes among CML patients vary between different studies, but also the impact of these subtypes on the phenotypic characteristics of CML patients is a conflicting issue (Balatzenko, Vundinti, & Margarita, 2011). What this study constitutes is the endeavor to find the principal transcript variants of *BCR/ABL* fusion gene in Iraqi CML patients; interrelation between these variants and their association with different aspects of the disease such as age, sex, hemoglobin, white blood cell count, and platelet count.

## 2 MATERIALS AND METHODS

This study was conducted over the period of September 2017 to June 2018. The study design was approved by the Institutional Review Board (Medical Ethics Committee) of the College of Medicine/ Al‐Nahrain University (No. 13/2017). An ethical clearance to conduct the research was also sought and obtained from the Iraqi National Center of Hematology for Research and Treatment/ Baghdad.

Clinical and laboratory data were obtained from patients’ records and database of the center. Peripheral blood samples were obtained for molecular analysis after informed consent from the patient.

### 2.1 Patients

One hundred consecutive chronic phase CML patients comprised 89 imatinib‐resistant and 11 newly diagnosed cases were enrolled in this study. They were 58 males and 42 females with age range 18–80 years (mean 51.7 ± 12.2 years). The diagnosis of CML was established on the basis of the peripheral blood parameters as well as morphological analysis of peripheral blood and bone marrow aspirates which were carried out in a single center (Iraqi National Center of Hematology for Research and Treatment/ Baghdad) and confirmed by the presence of Ph chromosome and/or fusion *BCR‐ABL* gene by conventional cytogenetics and reverse transcriptase polymerase chain reaction (RT‐PCR), respectively.

### 2.2 Materials

Blood samples (5 ml for each patient) were collected from peripheral blood into properly labeled ethylenediamine‐tetraacetic acid vacuum tubes. Processing of the collected blood, that is, ribonucleic acid (RNA) isolation from the collected blood sample, was started immediately in the laboratories of Medical Research Unit/ College of Medicine/ Al‐Nahrain University, in order to avoid any chance of messenger RNA degradation.

### 2.3 RNA isolation

Total RNA was isolated from a blood sample by using a ready commercial kit (GENEzol TriRNA Pure isolation kit/Geneaid/Taiwan) following the protocol provided by the manufacturer. A nanodrop (ACTGene/USA) was used to estimate the concentration and purity of the extracted RNA.

### 2.4 Complementary DNA synthesis

RNA was reverse transcribed into complementary DNA (cDNA) for using as template in PCR reaction. RT reaction was performed by using Accu Power Rocket Script RT PreMix (Bioneer/Korea). This kit is ready‐to‐use lyophilized master mix containing all components for first strand cDNA synthesis from RNA template.

### 2.5 Gene amplification and detection of fusion type

The cDNA was subjected to multiplex conventional PCR. Four primers were used for qualitative determination of *BCR‐ABL* fusion (Ujjan, Akhund, Saboor, Qureshi, & Khan, 2015) in the following sequences: *BCR*‐e1: 5′‐ACCGCATGTTCCGGGACAAAAG‐3’, *BCR*‐b2: 5′‐ACAGAATTCCGCTGACCATCAATAAG‐3′, *BCR*‐rev: 5′‐ATAGGATCCTTTGCAACCGGGYCYGAA‐3′ and *ABL*‐a2: 5′‐TGTTGACTGGCGTGATGTAGTTGCTTGG‐3′.

A ready master mix (Bioneer/Korea) of 20 µl was used for mixture preparation. Two microliter of template cDNA and 1 µl of each primer were added to the master mix tube. The final volume was adjusted to 20 µl with free nuclease distilled water. The mixture was then vortexed for 10 s and put in thermocycler (Bioneer/Korea) which was previously programmed with the following conditions: An initial denaturation for 5 min at 94°C followed by 40 cycles of denaturation at 94°C, annealing at 52°C and extension at 72°C all of which for 45 s. The final elongation was at 72°C for 5 min. The expected fragment length: 481 bp, ela2; 385 bp, b3a2; 310 bp, b2a2; 209 bp, b3a3 and 103 bp, b2a3 (Brinkmann, 2002). The PCR products were subjected to 2% agarose gel electrophoresis, stained with ethidium bromide and visualized with UV transilluminator.

### 2.6 Statistical analysis

The Statistical Package for the Social sciences (SPSS, version 20.0) was used for statistical analysis. Continuous variables were expressed as mean ± *SD*, and analyzed with Student's *t* test, while dichotomous variables were expressed as frequency and percentage, and analyzed with Chi‐squared test. All *p‐*values were two‐sided and the level of significance was taken as *p* < 0.05.

## 3 RESULTS

### 3.1 Demographic characteristics of the study population

The study included 100 patients, out of whom 58% were males and 42% were females (M:F ratio of 1.38:1) with a mean age of 51.7 ± 12.2 years (18–71 years). Out of 100 patients, 61% were younger than 45 years and the rest 39% were 45 years of age or older.

The BMI was used as a measure of fat tissue mass; 37% of patients had normal BMI, 54% were overweight and 9% were obese. More than two‐third of patients had either low or intermediate educational level, and reside in rural areas. Family history of hematopoietic cancer was reported in 11% of patients (Table 1).

**Table 1:** Baseline demographic characteristics of the study group

Variable		No (%)
Age (years)	<45	61 (61%)
	≥45	39 (39%)
Gender	Male	58 (58%)
	Female	42 (42%)
Body mass index (kg/m^2^)	<25	37 (37%)
	25–30	54 (54%)
	>30	9 (9%)
Educational level	Low	30 (30%)
	Intermediate	42 (42%)
	High	28 (28%)
Residence	Rural	69 (69%)
	Urban	31 (31%)
Family history	No	89 (89%)
	Yes	11 (11%)

### 3.2 Hematological characteristics of the study population

The blood count showed that Hb level, PCV and total RBC count were below the lower limit of normal range (Table 2). The mean corpuscular volume; MPV, mean platelet volume (MCV), mean corpuscular hemoglobin (MCH), mean corpuscular hemoglobin concentration (MCHC), RDW‐SD, and RDW‐CV were not different from normal values. Total WBC was higher than the standard values. The differential leukocyte count shows reduced neutrophil, normal lymphocyte and monocyte, raised eosinophil and basophil count. The total platelet count and its indices (plateletcrit [PCT], MPV, PDW‐SD, PDW‐CV) were within the approximation of normal ranges.

**Table 2:** Laboratory data of CML patients

Variable		Mean ± *SD*
Hemoglobin (g/dL)		12.28 ± 1.57
Packed cell volume %		35.45 ± 6.4
Red blood cell total count & indices	Total × 10^12^/L	3.6 ± 0.64
	MCV (fL)	90.13 ± 11.22
	MCH (pg)	31.25 ± 2.72
	MCHC (g/dL)	33.64 ± 3.91
	RDW‐SD (fL)	31.16 ± 4.86
	RDW‐CV %	11.44 ± 0.97
White blood cells	Total × 10^9^/L	45.26 ± 16.1
	Neutrophil %	44.85 ± 11.84
	Lymphocyte %	46.53 ± 8.37
	Monocyte %	9.22 ± 1.64
	Eosinophil %	4.03 ± 0.28
	Basophil %	4.83 ± 0.06
Platelets total count & indices	Total × 10^9^/L	341.5 ± 111.93
	PCT %	0.246 ± 0.92
	MPV (fL)	8.28 ± 1.33
	PDW‐SD (fL)	14.64 ± 1.98
	PDW‐CV %	31.17 ± 2.74

Abbreviations: CML, chronic myeloid leukemia; MCH, mean corpuscular hemoglobin; MCHC, mean corpuscular hemoglobin concentration; MCV, mean corpuscular volume; MPV, mean platelet volume; PCT, plateletcrit; PDW‐CV, coefficient of variation of platelet distribution width; PDW‐SD, standard deviation of platelet distribution width; RDW‐CV, coefficient of variation of red cell distribution width; RDW‐SD, standard deviation of red cell distribution width.

### 3.3 The frequency of different *BCR‐ABL* fusion variants

**Figure 1:**
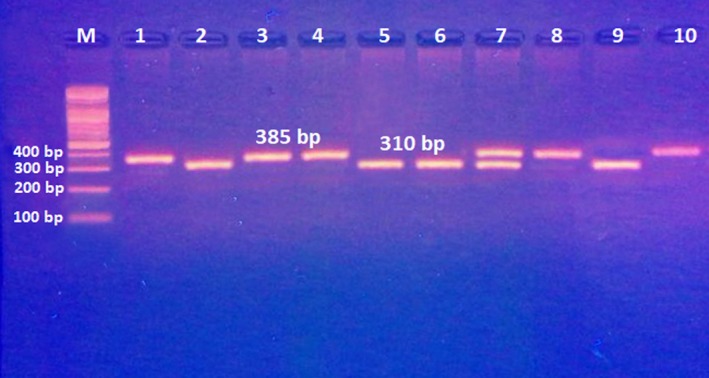
Gel electrophoresis of multiplex RT‐PCR products visualized under UV light after staining with ethidium bromide. M: 100–1500 bp DNA ladder; lanes 1, 3, 4, 8, and 10 represent b3a2 variant; lanes 2, 5, 6, and 9 represent b2a2 variants; lane 7 stands for a combination of b3a2 and b2a2 variant. RT‐PCR, reverse transcriptase polymerase chain reaction

Figure 1 shows gel electrophoresis of multiplex RT‐PCR products using four primers.

The primer combinations allowed reliable detection of typical p210 transcripts, such as b2a2 or b3a2, and atypical types, such as transcripts lacking *ABL* exon a2 (b2a3) simultaneously in one reaction.

The b3a2 transcript variant was recognized as 385 bp length product, b2a2 was recognized as 310 bp length product and b2a3 as 103 bp length product. Accordingly, 98 (98%) patients out of the total 100 expressed one of the P210*^BCR‐ABL^* rearrangements—b3a2 or b2a2.

**Figure 2:**
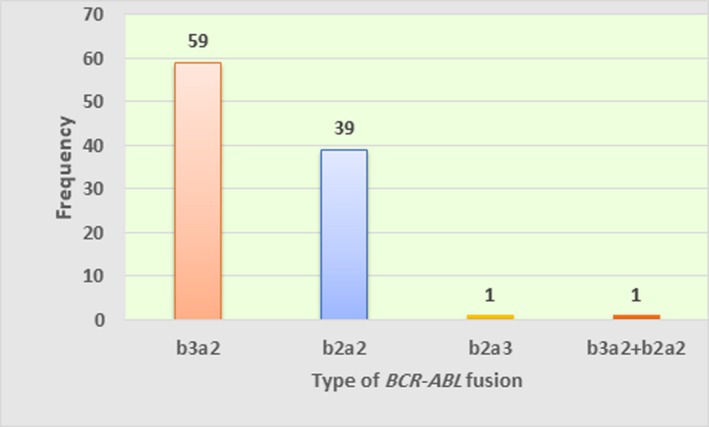
The Frequency of Different *BCR‐ABL* Fusion Variants

All studied patients were found to be M‐*BCR* positive and none with m‐*BCR* or µ‐*BCR*. Fifty‐nine (59%) of them expressed b3a2 transcript, whereas 39% of the remaining cases were positive for b2a2 variant. The rest 2% (numerically, 2 cases), one of them expressed b2a3 transcript, which is a rare variety of M‐*BCR/ABL* and another patient coexpressed two types of mRNA b3a2/b2a2 (Figure 2) and they were excluded from further analysis.

### 3.4 Demographic data based on *BCR‐ABL* fusion transcripts

The association of b3a2 and b2a2 transcripts with the patient's demographic data is presented in Table 3. Each of age, age at onset, BMI, educational level, residence, and family history had no significant association (*p* > 0.05).

**Table 3:** Patient's demographic data based on *BCR‐ABL* fusion transcript types

Variable No (%)		*BCR‐ABL* fusion variants		*p* value
		b3a2 *N* = 59	b2a2 *N* = 39	
Age (years)	<45	38 (64.41%)	23 (58.97%)	0.741
	≥45	21 (35.59%)	16 (41.03%)	
Age at onset (years)	<30	18 (30.51%)	9 (23.08%)	0.417
	≥30	41 (69.49%)	30 (76.92%)	
Gender	Male	40 (67.8%)	18 (46.15%)	0.022
	Female	19 (32.2%)	21 (53.85%)	
BMI (kg/m^2^)	<25	23 (38.98%)	13 (33.33%)	0.723
	25–30	31 (52.54%)	22 (56.41%)	0.707
	>30	5 (8.47%)	4 (10.26%)	0.766
Educational level	Low	17 (28.81%)	12 (30.77%)	0.836
	Intermediate	28 (47.46%)	13 (33.33%)	0.163
	High	14 (23.73%)	14 (35.9%)	0.194
Residence	Rural	41 (69.49%)	28 (71.79%)	0.807
	Urban	18 (30.51%)	11 (28.21%)	
Family history	No	51 (86.44%)	36 (92.31%)	0.365
	Yes	8 (13.56%)	3 (7.69%)	

Abbreviation: BMI, body mass index.

A significantly greater proportion of males (67.8%) compared to females (32.2%) present with high b3a2 variant expression. On the contrary, greater proportion of females (53.85%) compared to males (46.15%) present with b2a2 transcript expression (*p* = 0.022, Chi‐square test).

### 3.5 Hematological data based on *BCR‐ABL* fusion transcript types

Hematological characteristics of CML patient population stratified by *BCR‐ABL* transcripts are presented in Table 4. Differences in RBC count, PCV and Hb level were not significant (*p* > 0.05), yet, the values are below the minimum limit of normal in both (b2a2 and b3a2) transcripts.

**Table 4:** Patient's hematological data based on *BCR‐ABL* fusion transcript types

Variable	*BCR‐ABL* fusion variants		*p*‐Value
	b3a2 Mean ± *SD* *N* = 59	b2a2 Mean ± *SD* *N* = 39	
Packed cell volume %	36.18 ± 7.21	34.41 ± 7.8	0.115
Hemoglobin (g/dL)	12.84 ± 1.46	11.42 ± 2.06	0.408
Red blood cells × 10^12^/L	3.62 ± 0.71	3.57 ± 0.68	0.426
White blood cells × 10^9^/L	51.17 ± 19.7	36.32 ± 15.2	0.031
Neutrophil %	45.98 ± 12.63	43.13 ± 14.24	0.209
Lymphocyte %	43.79 ± 10.58	47.32 ± 9.22	0.273
Monocyte %	9.67 ± 1.71	8.41 ± 1.8	0.422
Eosinophil %	4.19 ± 0.37	3.81 ± 0.44	0.19
Basophil %	4.97 ± 0.08	4.62 ± 0.06	0.765
Platelets × 10^9^/L	296.4 ± 118.5	418.9 ± 221.2	0.041

Transcript‐stratified total leukocyte count was significantly different (*p* = 0.031, Student's *t* test), with b3a2 subtype showing higher count (51.17 ± 19.7 × 10^9^/L) than b2a2 subtype (36.32 ± 15.2 × 10^9^/L) WBC count. Differential leukocyte count showed a slight increase in the percent of eosinophils and basophils in both transcripts. However, there were no significant differences between the two transcripts with this regard (*p* > 0.05).

Additionally, patients with b2a2 subtype showed a significantly higher platelet count (418.9 ± 221.2 × 10^9^/L) compared to those with b3a2 transcript (296.4 ± 118.5 × 10^9^/L) (*p* = 0.041, Student's *t* test).

### 3.6 RBC and platelets indices based on *BCR‐ABL* fusion transcript types

Notable RBC and platelet indices of CML patient population stratified by *BCR‐ABL* transcripts are presented in Table 5. RBC indices (MCV, MCH, MCHC, RDW‐SD, and RDW‐CV %) were not significantly different between the two subtypes (*p* > 0.05). However, for most of these indices, patients with b2a2 transcript showed slightly higher values than those with b3a2 variant.

**Table 5:** Patient's RBC and platelets indices data based on *BCR‐ABL* fusion transcript types

Variable	*BCR‐ABL* fusion variants		*p*‐Value
	b3a2 *N* = 59 mean ± *SD*	b2a2 *N* = 39 mean ± *SD*	
Red blood cell Indices			
MCV (fL)	89.11 ± 12.76	91.72 ± 10.42	0.622
MCH (pg)	30.9 ± 2.93	31.79 ± 3.31	0.829
MCHC (g/dL)	32.61 ± 4.52	35.21 ± 4.44	0.208
RDW‐SD (fL)	31.95 ± 5.74	29.96 ± 4.46	0.342
RDW‐CV %	11.35 ± 0.96	11.58 ± 1.2	0.922
Platelets Indices			
PCT %	0.23 ± 0.09	0.28 ± 1.05	0.012
MPV (fL)	9.08 ± 2.04	7.04 ± 1.29	0.036
PDW‐SD (fL)	14.42 ± 2.09	14.97 ± 2.1	0.886
PDW‐CV %	30.54 ± 2.98	32.13 ± 3.78	0.773

Abbreviations: MCH, mean corpuscular hemoglobin; MCHC, mean corpuscular hemoglobin concentration; MCV, mean corpuscular volume; MPV, mean platelet volume; PCT, plateletcrit; PDW‐CV, coefficient of variation of platelet distribution width; PDW‐SD, standard deviation of platelet distribution width; RDW‐CV, coefficient of variation of red cell distribution width; RDW‐SD, standard deviation of red cell distribution width.

On the other hand, for platelet indices stratified by *BCR‐ABL* transcripts, the profiles were different. Significantly higher PCT percentage (*p* = 0.012, Student's *t* test) in those with b2a2 transcript as compared to those with b3a2 transcript (0.28% ± 0.05% vs. 0.23% ± 0.09) was observed. In contrast, MPV was significantly higher (*p* = 0.036, Student's *t* test) in CML patients with b3a2 transcript than those with b2a2 variant (9.08 ± 2.04 ft vs. 9.08 ± 2.04 ft).

## 4 DISCUSSION

In this study, the frequency of M‐*BCR* transcript variants (b3a2 and b2a2) was found to be 59% and 39%, respectively. The coexpression of b3a2/b2a2 transcripts was found in one patient. Another patient expressed rare b2a3 variant. To the best of our knowledge, this is the first study in our country which denotes the frequency of *BCR‐ABL* fusion transcripts.

Data of multiple studies have been published about the frequency of these *BCR‐ABL* fusion oncogenes associated with CML in different population with controversial results (Table 6).

**Table 6:** Incidence of b2a2 and b3a2 transcripts in Eastern, African, European, and Western countries

Country	No. of patients	*BCR‐ABL* fusion gene	
		b3a2%	b2a2%
Present study	100	59	39
India (Kagita et al., 2018)	170	63.53	36.36
Iran (Ayatollahi et al., 2018)	85	62.35	29.41
Syria (Al‐Achkar et al., 2016)	45	51.1	46.7
Pakistan (Irshad, Butt, & Joyia, 2012)	23	26	65
Saudi Arabia (Iqbal, 2014)	30	63.33	36.66
Korea (Goh et al., 2006)	548	67.66	32.34
Japan (Ito et al., 2004)		67.9	30.2
Germany (Hanfstein et al., 2014)	1,105	44.89	40.81
Bulgaria (Balatzenko et al., 2011)	98	54	45
UK (Lucas et al., 2009)	71	39	31
Brazil (Vasconcelos et al., 2017)	203	64	34
USA (Jain et al., 2016)	481	41	42
Argentine (Sastre et al., 2007)	24	37.5	41.7
Ecuador (Paz‐y‐Mino et al., 2002)	40	5.4	94.6
Mexico (Arana‐Trejo et al., 2002)	250	35	48
Tunisia (Bennour et al., 2013)	45	64	36
Sudan (Osman et al., 2010)	46	41.9	53.5

In the vast majority of aforementioned studies from the Far‐East to the Far‐West, frequency of b3a2 transcript was higher than b2a2 transcript in CML patients. The present study is one of few series that examines relatively large number of CML patients.

The differences in the data of these series could be ascribed to four reasons. First of all, the ethnicity of the study population; different population reasonably have different frequency of certain transcript due to different genetic background. Secondly is probably the geographical distribution. Thirdly, the sample size; the larger is the sample size the more it is representative and reproducible results. Finally, the sampling time; obtaining blood samples form newly diagnosed patients is more accurate than do during imatinib treatment (even with the presence of imatinib resistance), as this tyrosine kinase inhibitor (TKI) found to affect the rare transcripts like e1a2 (Verma et al., 2009), while the coexpression of b3a2 and b2a2 may present at initial stage, but as the disease progress only one of them would prevail (Leglise et al., 1996).

Gender was the only demographic feature that showed significant association with *BCR‐ABL* fusion type as illustrated in Table 3. Worldwide data considering this association demonstrate contradictory findings. This study revealed the predominance of b3a2 transcript in males compared to females whereas b2a2 transcript was more common in females.

In Mexico, Arana‐Trejo et al. (2002) studied 226 CML adult patients and labeled proportions of male to female with b3a2 and b2a2 transcripts into 44:34% and 48:59%, respectively. Also, in harmony with the current findings, Kagita et al. (2018) investigated 170 Indian patients with CML for *BCR‐ABL* fusion types. They reported a prevalence of 66.05% and 54.09% in male and female patients expressing b3a2, respectively compared to 33.02% and 39.3% for male and female patients with b2a2 transcript, respectively.

However, Al‐Achkar, Moassass, Youssef, and Wafa (2016), Hanfstein et al. (2014) and Hamid and Bokharaei (2017) did not catch any association between gender and *BCR‐ABL* transcripts. Furthermore, other studies registered reversed setting, that is, b3a2 transcript was more common in females and b2a2 transcript was more common in males. In Saudi Arabia, Mir et al. (2015) recruited 200 patients with CML and found that male gender was significantly associated with b2a2 transcript. Similarly, Adler et al. (2009) revised 146 German pediatric patients with CML and reported 34 males (51%) versus 21 females (27%) with b2a2 transcript and 17 males (25%) versus 36 females (45%) with b3a2 variant.

In Sudanese, a significant correlation between sex and transcript type was noticed, in which male patients showed a higher tendency of expressing b2a2 and females patients showed a higher tendency for b3a2 (Osman, Hamad, Elmula, & Ibrahim, 2010).

It seems that the ethnicity of the study population, sample size, and method of detection could be responsible for such variations.

How gender can influence the type of *BCR‐ABL* fusion transcript is a matter of debate. Some neuroendocrinological studies approved hormonal‐related splicing in rat and mice (Thakur & Mani, 2005) and claimed that similar manner may occur in human which influences the splicing of *BCR‐ABL* gene and largely determines the type of transcript. Another proposed mechanism is the association of angiotensin‐converting enzyme intronic insertion/deletion polymorphism with the type of transcript. This polymorphism is known to have an influence on gene expression and transcript splicing (Hull et al., 2007). Notably, there is a high difference in the incidence of this polymorphism between males and females with a possible effect on *BCR‐ABL* fusion subtypes.

The association between *BCR‐ABL* fusion variants was dealt with by many researchers worldwide. Many studies revealed a significant increase in WBC count in CML patients with b3a2 transcript and high platelet count in those with b2a2 variant. Few studies find almost the opposite, that is, high leukocyte and platelet count in those with b2a2 and b3a2 transcript, respectively. Still more other studies found no differences in this regard. Table 7 shows the main published articles dealt with laboratory data and *BCR‐ABL* transcript.

**Table 7:** Main published studies about the association between p210*^BCR‐ABL1^* transcript types and laboratory data in patients with CML

Study	*BCR‐ABL* fusion gene transcripts			
	b3a2		b2a2	
	WBC	Platelet	WBC	Platelet
Present study	↑			↑
Kagita et al. (2018)	↑	↑		
Balatzenko et al. (2011)		↑		
Ayatollahi et al. (2018)	nd		nd	
Al‐Achkar et al. (2016)		↑	↑	
Hanfstein et al. (2014)	↑			↑
Vasconcelos et al. (2017)		↑	↑	
Bennour et al. (2013)		↑		
Deb et al. (2014)	↑			↑
Polampalli et al. (2008)	nd		nd	
Rosas‐Cabral et al. (2003)	nd	↑	nd	
Perego et al. (2000)		↑		
Inokuchi et al. (1991)		↑		

Abbreviation: CML, chronic myeloid leukemia; nd, no difference.

This variability in the correlation between specific fusion type and the WBC or platelet count was explained by many authors. One group attributed this to the conformational difference between the two transcripts as b3a2 transcript is longer than b2a2 transcript by 25 amino acids in the *BCR* region. Although this region does not have a tyrosine kinase activity, the extra 25 amino acid residues may change the overall conformation of *BCR‐ABL* fusion protein in a way that reduces the kinase activity (Lin et al., 2016). Nevertheless, this explanation is not satisfactory in the current study because b3a2 transcript correlated with increased total WBC count and b2a2 transcript correlated with increased platelets count.

Other group relates this variability to the effect of b3a2 transcript on the thrombopoietic activity (Inokuchi et al., 1991), which is also not appropriated for the current results.

Alternatively, the most plausible explanation is that many signaling pathways, other than tyrosine kinase, are involved in leukopoietic and thrombopoietic process, and any defect in one or more pathways may associate with disturbance of overall hematopoietic process. Supporting this hypothesis is the study by Balatzenko et al. (2011) who investigated the impact of *BCR‐ABL* fusion types in association with multi‐drug resistance (*MDR1*) gene on hematological parameters in 89 Indian patients with CML. The human *MDR1* gene encodes an integral membrane protein, P glycoprotein (Pgp), whose function is the energy dependent export of substances from the inside of cells, and from membranes, to the outside. Its physiological role is the protection of cells from toxic substances or metabolites (Brinkmann, 2002). Interestingly, the study showed significant correlation between b3a2 and an increment in platelet count only in patients who had overexpression of *MDR1* gene. The authors attributed this increase to the presence of common signaling cascade between b3a2 transcript and *MDR1* gene.

Based on this finding, it is reasonable to assume other signaling pathways that influence the bone marrow and interfere with kinase activity of b3a2 and b2a2 variants, and the significant correlation between each transcript with a certain hematological parameter should be carefully explained.

With regard to RBC indices, they were comparable between b3a2‐ and b2a2‐expressing patients with no significant differences. On the other hand, platelets indices show significant variation between the two groups. CML patients with b3a2 variant demonstrate higher percentage of PCT as compared to those with b2a2 variant and on the reverse for MPV (low in b3a2 and high in b2a2 CML patients). Retrieval of the available international studies denotes no such findings (Ayatollahi et al., 2018). In fact, the relative decrease in PCT% in CML patients expressing b3a2 cannot be attributed to the transcript itself; rather the relative low platelets count is the main factor in this case, that is, the lower the platelets count, the lower their cumulative volume (PCT%). Similarly, the relative decrease in MPV in CML patients with b2a2 transcript could be explained on the same base, that is, the higher the platelets number, the smaller the individual volume of each platelet.

In conclusion, all studied Iraqi Philadelphia‐positive CML patients expresses the M‐*BCR‐ABL* subtype of the fusion gene; a preponderance of b3a2 over b2a2 variant; a sex‐skewed distribution in *BCR‐ABL* transcript types with b3a2 transcript is more prevalent in males and b2a2 subtype in females and the type of *BCR‐ABL* transcript is reflected by different leukocyte and platelet counts, which might represent a distinct phenotype and disease biology.

We recommend identifying the *BCR‐ABL* transcript type in every CML patient at diagnosis along with the cytogenetic study, linking the detected transcript with the patient's clinical findings, possible resistance to first‐line TKI.

## CONFLICTS OF INTEREST

The authors declare that they have no conflicts of interest.

## AUTHOR CONTRIBUTIONS

All the authors have directly participated in the preparation of this manuscript and have approved the final version submitted. Khazaal contributed the collection of cases. Al‐Mayah performed the statistical analysis. Khazaal drafted the manuscript. Al‐Mayah and Hamdan conceived the study and participated in its design and interpretation. Al‐Mayah and Hamdan supported the manuscript drafting. All the authors have read and approved the final manuscript.
